# Determination of Sulfonamides in Feeds by High-Performance Liquid Chromatography after Fluorescamine Precolumn Derivatization

**DOI:** 10.3390/molecules24030452

**Published:** 2019-01-28

**Authors:** Ewelina Patyra, Monika Przeniosło-Siwczyńska, Krzysztof Kwiatek

**Affiliations:** Department of Hygiene of Animal Feedingstuffs, National Veterinary Research Institute, Partyzantów 57 Avenue, 24-100 Puławy, Poland; Monika.Przenioslo@piwet.pulawy.pl (M.P.-S.); kwiatekk@piwet.pulawy.pl (K.K.)

**Keywords:** sulfonamides, non-target feed, HPLC, FLD, method validation, SPE, Strata-SCX, 657/2002/EC

## Abstract

A new multi-residue method for the analysis of sulfonamides (sulfadiazine, sulfamerazine, sulfamethazine, sulfaguanidine and sulfamethoxazole) in non-target feeds using high-performance liquid chromatography-fluorescence detection (HPLC-FLD) and precolumnderivatization was developed and validated. Sulfonamides (SAs) were extracted from feed with an ethyl acetate/methanol/acetonitrile mixture. Clean-up was performed on a Strata-SCX cartridge. The HPLC separation was performed on a Zorbax Eclipse XDB C18 column with a gradient mobile phase system of acetic acid, methanol, and acetonitrile. The method was validated according to EU requirements (Commission Decision 2002/657/EC). Linearity, decision limit, detection capability, detection and quantification limits, recovery, precision, and selectivity were determined, and adequate results were obtained. Using the HPLC-FLD method, recoveries were satisfactory (79.3–114.0%), with repeatability and reproducibility in the range of 2.7–9.1% to 5.9–14.9%, respectively. Decision limit (CCα) and detection capability (CCβ) were 197.7–274.6 and 263.2–337.9 µg/kg, respectively, and limit of detection (LOD) and limit of quantification (LOQ) were 34.5–79.5 and 41.3–89.9 µg/kg, respectively, depending on the analyte. Results showed that this analytical procedure is simple, rapid, sensitive, and suitable for the routine control of feeds.

## 1. Introduction

During the last decades, livestock production has increased markedly, mainly due to intensive farming. Veterinary medicines are extensively used in animal husbandry in order to treat bacterial infections as well as for prophylactic purposes. One group commonly used as antibacterial drugs in both human and veterinary medicine is sulfonamides (SAs). Sulfonamides are synthetic antimicrobial compounds that are widely used to treat respiratory, gastrointestinal, and urinary tract infections [1]. Sulfonamides are high-spectrum chemotherapeutics against Gram-positive and Gram-negative bacteria and are used for the treatment of infections caused by microorganisms resistant to other antibiotics [2]. High doses of SAs may provoke strong allergic reactions, hence the medicines are prescribed carefully. In veterinary practice, SAs can be incorporated into animal feed (medicated feed) as a therapeutic agent. In unmedicated feeds, SAs can be present because traces of previously manufactured medicated feed may be accidentally mixed with the first batches of the next feed when the same production line is used. The transfer of traces of compounds from one manufactured batch of feed to the next is called carry-over. Consequently, current feed production technologies may lead to the unavoidable cross-contamination of unmedicated feeds with undesirable substances, contributing possible threats to animal health and public health.

In the European Union, animal feeds must fulfill several rules laid down by current legislation [3,4,5]. The main rules give requirements for the composition, storage, transport, and usage of animal feeds. Cross-contamination can occur during production and handling in the feed mill, during transport, or on the farm. Carry-over of veterinary drugs during feed production may also cause the contamination of non-medicated feedstuffs. The use of antibiotics in feed for non-medicinal purposes was banned in the EU in 2006. Monitoring for the undeclared or illegal use of these antibacterial substances is conducted within national feed control programs [6].

Several analytical methods have been developed for the identification and quantification of sulfonamide residues in animal tissues, eggs, milk, and honey [7,8,9,10,11]. Existing methods include high-performance liquid chromatography (HPLC) with different detectors: diode array/UV detector [1,12,13,14], fluorescence [15], mass spectrometry [9,16,17], gas chromatography (GC) [7], and capillary electrophoresis (CE) [2]. However, for the analysis of sulfonamides in medicated feed and non-target feed, only a few methods are described in the literature. The methods of detection and determination of sulfonamides in feed are based on liquid chromatography with UV detector [1], diode array detector [14], and enzyme-linked immunosorbent assay test (ELISA) [10]. Liquid chromatography tandem mass spectrometry (LC-MS/MS or UHPLC-MS/MS) methods have been used for the validation of multi-residue quantification of sulfonamides in feeds [18,19,20,21,22].

Kim et al. developed a HPLC-UV method for the determination of sulfacetamide, sulfadiazine, sulfathiazole, sulfapyridine, sulfamerazine, sulfamethizole, sulfamethazine, sulfachloropyridazine, sulfamethoxazole, sulfamonomethoxine, sulfisoxazole, sulfadimethoxine, sulfaquinoxaline, sulfamethoxydiazine, sulfisomidine, and sulfachloropyrazine in feed. Recoveries obtained were in the range of 78.2–105.2%, whereas limit of quantification (LOQ) values ranged from 46.9–150.0 µg/kg [1]. 

A method for the determination of sulfadiazine and sulfamethazine in feed was developed by Patyra et al., who used an LC-MS/MS instrument. Average recovery was in the range of 76.0–77.4%, and LOQ values ranged from 106.2–174.6 µg/kg [21].

Gavilán et al. proposed a method to determine sulfaclorpiridazine, sulfadiazine, sulfametazine, sulfamethizol, sulfametoxazol, sulfametoxipiridazine, sulfapiridazine, sulfaquinoxaline, sulfatiazol, and sulfadimidine in feed samples with the use of LC-MS/MS, with a mean recovery of 84–113% [18].

Liu et al. proposed a method to determine 16 sulfonamides in feed with the use of a solid phase extraction (SPE) technique with basic alumina cartridges and LC-MS/MS, with a mean recovery of 80.1–111.7% [20].

In this study, liquid chromatography with fluorescence detector after precolumn derivatization with fluorescamine for the detection and quantification of five sulfonamides in non-target feed is used for the first time. This paper presents the development of a selective and sensitive method for the simultaneous analysis of five SAs using a strong cation exchange Strata-SCX cartridge extraction, fluorescamine derivatization, and high-performance liquid chromatography-fluorescence detection(HPLC-FLD) analysis. The whole procedure was validated in accordance with the Commission Decision 2002/657/EC [23].

## 2. Results and Discussion

### 2.1. Sample Preparation 

Feed samples are considered difficult matrices due to their variable and complex composition, which is why the choice of an appropriate extraction solvent and good purification strategy is a crucial issue.

As described in the literature, good recoveries of SAs from pig, poultry, horse, and cattle feeds have been obtained when extraction was carried out with organic solvents: acetonitrile or methanol with or without water [1,17,19,21,22]. However, the use of methanol or acetonitrile for the extraction of SAs from a feed matrix results in a highly contaminated extract that makes the detection of low levels of SAs impossible.

**Figure 1:**
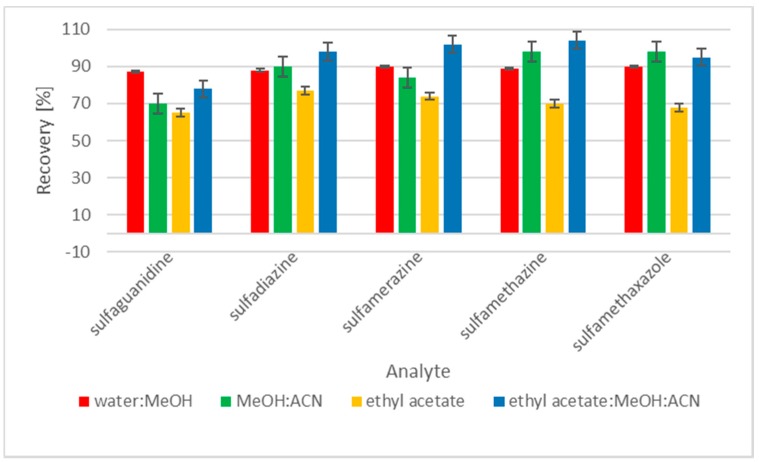
Recovery of sulfonamides employing different extraction solvents (*n* = 6).

In this study, four different extraction protocols for SAs in feed were tested. First, we tested the extraction protocol described by Kim et al. [1]. Sulfonamides were extracted with the use of a water/methanol mixture (20:80 *v*/*v*). The second option was the application of a mixture of methanol and acetonitrile (50:50 *v*/*v*) [17]. The next option for the extraction of SAs from a feed matrix was the use of ethyl acetate and a mixture of ethyl acetate/methanol/acetonitrile (50:25:25 *v*/*v*/*v*) (Figure 1). The final selected option was the use of a mixture of ethyl acetate/methanol/acetonitrile (50:25:25 *v*/*v*/*v*) for the extraction of five sulfonamides from the feed. The extracts required better preparation of the sample. Two variants of purification were investigated to effectively eliminate endogenous substances that were coextracted and interfered with the determination of sulfonamides. Solid phase extraction was used with Oasis HLB and Strata-SCX cartridges. First, supernatant from ethyl acetate/methanol/acetonitrile (50:25:25 *v*/*v*/*v*) was dried under nitrogen, reconstituted in Milli-Q water, and then introduced into an Oasis HLB cartridge. The Oasis HLB column was preconditioned by passing 3 mL of methanol and 3 mL of Milli-Q water. The analyte was eluted with 3 mL of methanol. For the Strata-SCX column, supernatant from ethyl acetate/methanol/acetonitrile extraction was directly transferred into the preconditioned column. Strata-SCX cartridges, which were prepared by using 5 mL of 40% acetic acid in acetonitrile for conditioning, were loaded with 6 mL of the extract, and interfering substances were eluted using 2.5 mL of acetone, 2.5 mL of methanol, and 2.5 mL of acetonitrile. Next, the supernatant was evaporated and residues were resuspended in 0.2% fluorescamine in acetone and 0.1 M sodium acetate pH=3.5. SAs were derivatized for 15, 30, and 45 min in the dark and at room temperature.

**Figure 2:**
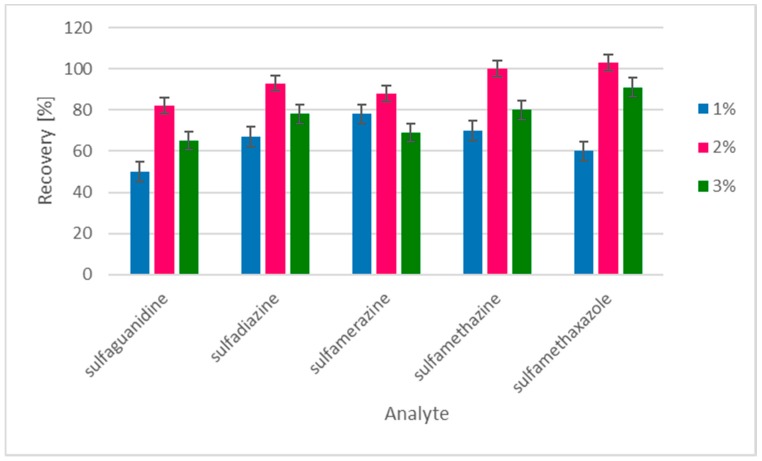
The recoveries of 5 sulfonamides with three SPE eluting conditions (*n* = 6).

The clean-up process using Strata-SCX cartridges was further optimized by using three different concentrations of ammonium solution in acetonitrile (1%, 2% and 3%). Three SA-spiked feeds (200, 1000, and 2000 µg/kg) were tested to compare the recoveries of different eluents. Results are shown in Figure 2. The experiments showed that the best recoveries were obtained with the use of Strata-SCX cartridges, and SAs were eluted using 2% ammonium solution in acetonitrile. The optimal time for sulfonamide derivatization was 15 min.

### 2.2. Chromatographic Conditions

Only a few analytical procedures have been described for the determination of SA residues in pig, poultry, cattle, and horse feeds. Ultraviolet detection is used for the detection and quantification of SAs in medicated feeds, but this technique is not suitable for non-target feed because of its lack of sensitivity and selectivity. For the detection of low concentrations of sulfonamides in non-target feed, liquid chromatography with atmospheric pressure chemical ionization and mass spectrometry (APCI-MS/MS) [12] or electrospray ionization with mass spectrometry (ESI-MS/MS) [19] are applied.

To improve the separation, sensitivity, and selectivity of the selected analytes, chromatographic conditions were optimized. For the analysis of SAs in feed, scientists often use as a mobile phase acetic acid or formic acid in Milli-Q water in combination with acetonitrile, or methanol with or without acetic acid or formic acid. For the chromatographic analysis of 16 sulfonamides in feed, Kim et al. [1] used 0.1% acetic acid in phosphate buffered saline (PBS) and 0.1% acetic acid in methanol for high performance liquid chromatography with diode array detector (HPLC-DAD) analysis. For the separation of SAs, Pereira-Lopes et al., Patyra et al., and Gavilán et al. used 0.1% formic acid in water, 0.1% formic acid in acetonitrile, and LC-MS/MS detection [17,19,22]. 

For the separation of sulfonamides, researchers have used C12 or C18 LC columns such as Hypersil RP C18, Luna C18, Unision UK-C18, Mediterranean Sea C18, C12 Phenomenex Hydro-RP, Kinetex biphenyl, or Synergi Polar RP columns [1,2,14,17,19,20,22]. 

**Figure 3:**
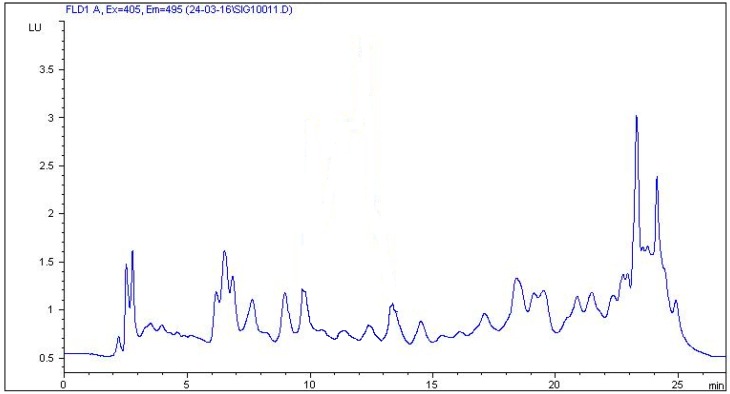
Chromatogram of blank feed sample.

**Figure 4:**
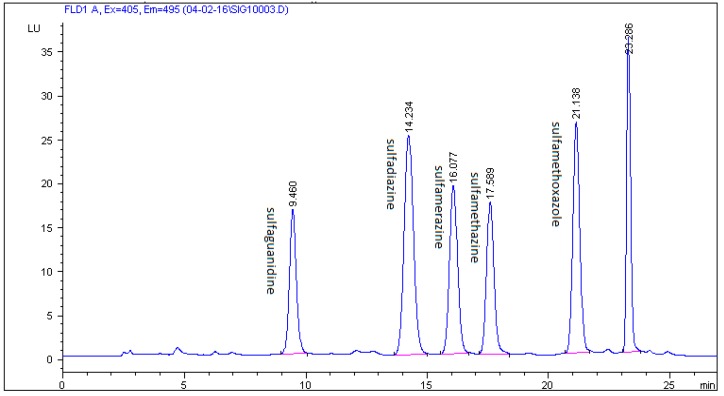
Chromatogram of feed sample spiked with five SAs at a concentration of 200 µg/kg on a Zorbax Eclipse XDB C18 column with a mobile phase of 0.08% acetic acid in Milli-Q water/acetonitrilemethanol.

In this study, a combination of three mobile phases including methanol, acetonitrile, 0.1% formic acid, 0.1% acetic acid, and 0.08% acetic acid in Milli-Q water and two different C18 chromatographic columns (Zorbax Eclipse XDB and Kinetex C18) were investigated. The best results were achieved using 0.08% acetic acid in Milli-Q water, methanol, and acetonitrile with a gradient elution and a Zorbax Eclipse XDB C18 chromatographic column. The selected multistep gradient elution was the result of a number of different elution programmes trying to yield optimum separation of the five studied SAs in 27 min. Retention times of the examined analytes were 9.460 min for sulfaguanidine, 14.234 min for sulfadiazine, 16.077 min for sulfamerazine, 17.589 min for sulfamethazine, and 21.138 min for sulfamethoxazole. Typical chromatograms of blank and spiked feed samples are shown in Figure 3 and Figure 4. The unknown peak at 23.286 min from feed matrix was well resolved from analytes. According to the authors’ knowledge, the presented method is the first to be described for the detection and quantification of five sulfonamides in non-target feed using HPLC and fluorescence detector. 

**Figure 5:**
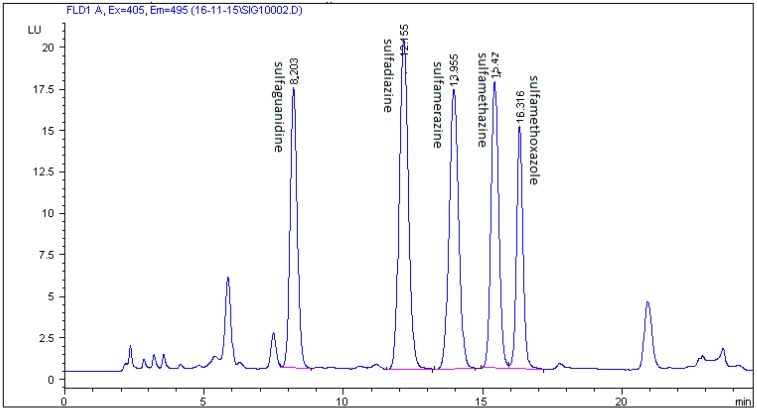
Chromatogram of feed sample spiked with five SAs at a concentration of 200 µg/kg on a Zorbax Eclipse XDB C18 column with a mobile phase of 0.1% acetic acid in Milli-Q water/acetonitrile/methanol.

**Figure 6:**
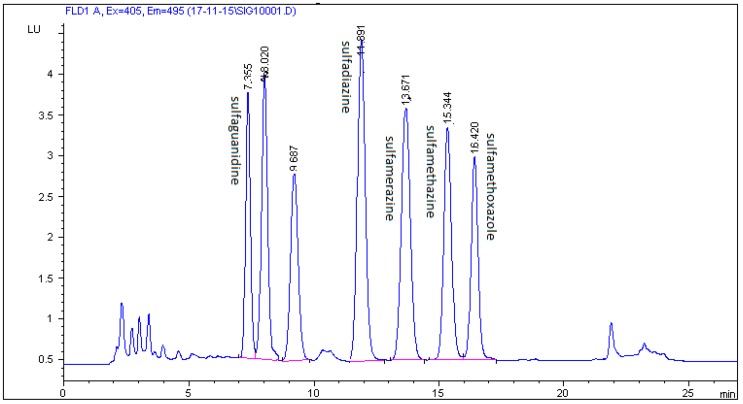
Chromatogram of feed sample spiked with five SAs at a concentration of 200 µg/kg on a Zorbax Eclipse XDB C18 column with a mobile phase of 0.1% formic acid in Milli-Q water/acetonitrile/methanol.

Figure 4, Figure 5 and Figure 6 show a comparison of separation effects between the three different mobile phases tested on the Zorbax Eclipse XDB column. 

### 2.3. Method Validation

In light of the lack of guidelines related to the validation protocol for the detection of antimicrobials in feed by HPLC-FLD, a validation protocol was established to prove that method performance was fit for the purpose, taking into account the requirements of Commission Decision 2002/657/EC [23]. The evaluated parameters were linearity, selectivity, specificity, sensitivity, repeatability, reproducibility, limit of detection (LOD), limit of quantification (LOQ), decision limit (CCα), detection capability (CCβ), and uncertainty.

Values for recoveries of the spiked samples were in the range of 79.3–114.0% for all analyzed sulfonamides. The intra-day and inter-day precision of the methods were evaluated at three concentration levels (200, 1000, and 2000 µg/kg), in line with the EU Commission Decision. For this purpose, six spiked samples at each level were prepared and analyzed. This procedure was repeated for three days in order to determine the inter-day precision. The repeatability and within-laboratory reproducibility for the target analytes were lower than 6% and 15%, respectively, at all spiking levels. Kim et al. [1] have developed a method for analyzing 16 sulfonamides with recoveries in the range of 78.2 to 105.5%, but they used immunoaffinity chromatography and HPLC-UV. Iammarino et al. [16] have developed a method for the detection of ten sulfonamides (sulfadiazine, sulfathiazole, sulfamerazine, sulfamethazine, sulfachloropyridazine, sulfamethoxazole, sulfaquinoxaline, sulfadimethoxine, sulfamonomethoxine, and sulfadimethoxine) and obtained recoveries ranging from 86.4% to 100.5% for all analyzed substances.

In the describedmethod, both LOD and LOQ values were determined. The LOD for the sulfonamides was 34.5–79.5 μg/kg, while the LOQ was 41.3–89.9 μg/kg. For 10 sulfonamides in feed, Iammarino et al. [16] obtained LOD and LOQ values of 390–640 μg/kg and 1290–2130 μg/kg, respectively. For the method we developed, CCα and CCβ values were 197.7–274.6 μg/kg and 239.2–337.9 μg/kg, respectively. Matrix effects were ±35%, which is in compliance with SANTE/11945/2015 requirements [24]. The expanded uncertainty was estimated to be in the range of 19.8–24.4%, depending on the analyte. All validation parameters are shown in Table 1. 

### 2.4. Real Sample Application

**Figure 7:**
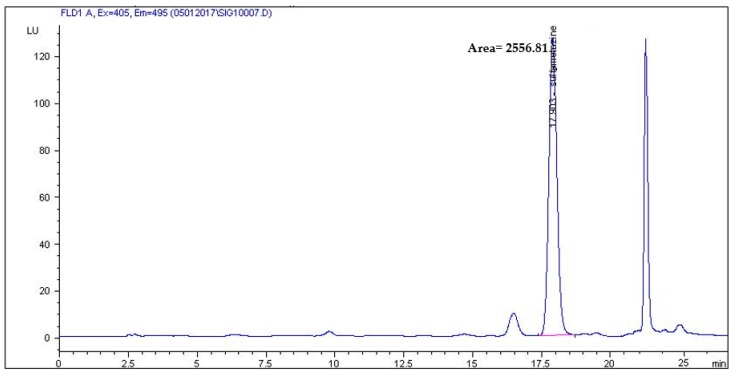
Chromatogram of feed sample that contained 1548 μg/kg of sulfamethazine.

The validated method was applied to the analysis of six poultry and swine feed samples. In one sample, sulfamethazine was detected at a concentration of 1548 µg/kg. The determined level of sulfadiazine reported byGavilán et al. was from 50 to 304 µg/kg [18]. These results are in agreement with the data reported by Croubels, who measured sulfadiazine in 27% of feed samples [12]. Patyra et al. detected sulfadiazine in three feed samples at concentrations of 250–2960 µg/kg [21]. Kim et al. analyzed 156 animal feeds and detected the presence of SAs in feeds that were used on farms, but not the ones that were purchased from markets. SAs were detected in four different kinds of animal feeds: bovine, pork, chicken, and duck. Sulfamethoxazole and sulfamethazine were found in concentrations of 150–155 µg/kg and 161–468 µg/kg, respectively [1]. Therefore, the indiscriminate use of SAs as additives in animal feeds must be stopped by government regulation, as well as by maximum residue limit (MRL)standards for the proper amount of SAs. Figure 7 presents an example chromatogram of a feed sample with sulfamethazine.

## 3. Materials and Methods

### 3.1. Chemicals and Reagents

Sulfadiazine (SDZ), sulfaguanidine (SGD), sulfamethazine (SMZ), sulfamerazine (SMR), sulfamethoxazole (SMO), and fluorescamine were obtained from Sigma Aldrich (St. Louis, MO, USA). HPLC-grade acetonitrile, methanol, and acetone were purchased from Baker (Deventer, The Netherlands). Ethyl acetate, acetic acid (99.5%), and sodium acetate were purchased from Chempur (PiekaryŚląskie, Poland), and the ammonia solution 25% was purchased from POCH (Gliwice, Poland). Purified water was prepared in-house with a Milli-Q water system from Millipore (Bedford, MA, USA).

### 3.2. Instrumentation

For sample preparation, a vortex mixer (Select BioProducts, NJ, USA), laboratory shaker (Gerhardt Analytical Systems, Königswinter, Germany), and laboratory centrifuge (Sigma, Taufkirchen, Germany) were used. The chromatographic system consisted of an Agilent 1100 HPLC system (Santa Clara, CA, USA) equipped with a quaternary pump, vacuum degasser, automatic injector, column thermostat, diode array, and fluorescence detector, and integration with ChemStation software. An SPE manifold (J.T. Baker, Arnhem, the Netherlands) and pump were used in the purification protocol with two different SPE cartridges: Strata SCX (500 mg, 3 mL) from Phenomenex (Torrance, CA, USA) and Oasis HLB cartridges (60 mg, 3 mL) from Waters (Milford, MA, USA), which were tested.

### 3.3. Chromatography

The separation of the sulfonamides was performed on a Zorbax Eclipse XDB (150 × 4.6 mm, 5 µm) column from Agilent Technologies (Santa Clara, CA, USA) protected by a RP18 guard column (4.0 × 3.0 mm, 5 μm) from Phenomenex (Torrance, CA, USA). The gradient was applied with 0.08% acetic acid in Milli-Q water (phase A), acetonitrile (phase B), and methanol (phase C). The gradient is shown in Table 2. The flow rate was 0.6 mL/min, and the injection volume was 40 μL. The column temperature was 25 °C. The excitation and emission wavelengths for all analyzed sulfonamides were 405 and 495 nm, respectively.

### 3.4. Standard Solutions

Standard stock solutions of individual sulfonamides (1 mg/mL) were prepared in methanol for sulfaguanidine, sulfamethazine, sulfamerazine, and sulfamethoxazole. A sulfadiazine standard was prepared by dissolving in acetonitrile. Sulfonamide working solutions of 100 μg/mL were prepared by dilution of the stock solutions in methanol and were stored in dark glass bottles at −18 °C for less than 6 months. A fluorescamine solution was prepared by weighting 20 mg of standard and dissolving in 5 mL of acetone. The fluorescamine solution was stored in a dark glass bottle at −18 °C for less than 3 months.

### 3.5. Sample Preparation

Previously ground poultry and pig feed samples of 5 g ± 0.01 g were transferred into 50 mL polypropylene centrifuge tubes and prepared by adding appropriate volumes of sulfonamide working solutions. After vortexing for 30 s, the feed samples were kept at room temperature for 60 min to enable sufficient equilibration with the feed matrix. Then, 20 mL of extraction mixture consisting of ethyl acetate/methanol/acetonitrile (50:25:25 *v*/*v*/*v*) was added and the content of the tubes was shaken at room temperature for 30 min on a horizontal shaker and centrifuged at 3500 rpm for 10 min at 20 °C.

### 3.6. Clean-Up

For the clean-up step, the SPE apparatus and Strata-SCX cartridges (500 mg, 3 mL) were used. Prior to sample loading, the cartridges were preconditioned with 5 mL of 40% acetic acid in acetonitrile. After percolation, the cartridges were washed with 2.5 mL of acetone, 2.5 mL methanol, and 2.5 mL of acetonitrile. The analytes were eluted with 2 × 2.5 mL of a mixture of 2% of ammonium solution in acetonitrile. The eluate extract was evaporated to dryness under a nitrogen stream at 40 °C ± 5 °C.

### 3.7. Derivatization

For FLD detection, dry residue was resuspended in 800 µL of acetate buffer (pH = 3.5). Then, 200 µL of the fluorescamine reagent was added and the solution was mixed with a vortex mixer. The sample was ready to analyze after standing for 15 min at ambient temperature in a dark place.

### 3.8. Validation Procedure

The proposed HPLC-FLD method was validated by a set of parameters that are in compliance with the recommendations defined by the European Commission Decision 2002/657/EC and ICH guidelines. The linearity of the method was evaluated using fortified blank feed samples. Good linearity was achieved by the analysis of feed samples spiked with standard solutions in the range of 200–2000 µg/kg, with correlation coefficients higher than 0.995 for all analyzed sulfonamides. The LOD, LOQ, CCα, and CCβ parameters were estimated using the calibration curve procedure. The limit of detection (LOD) is the lowest concentration of analyte that the analytical process can reliably differentiate from background levels, while the limit of quantification (LOQ) is the lowest concentration of analyte that can be quantified. LOD and LOQ values were calculated from a signal-to-noise ratio (S/N) of 3 and 10, respectively. CCα was calculated by analyzing 20 blank feed samples. A matrix-matched calibration curve was prepared, and the decision limit (CCα) and detection capability (CCβ) were determined according to the European Commission Decision 2002/657/EC for substances with non-permitted limits. CCα was calculated with a statistical certainty of 1 − α (α = 1%), whereas CCβ was calculated with a statistical certainty of 1 − β. CCβ was calculated as the decision limit plus 1.64 times the corresponding standard deviation (β = 5%). The selectivity/specificity of the method was tested by analyzing 20 blank feed samples to verify the absence of potential interfering endogenous compounds at the target analyte retention times. Intra-day precision was assessed by comparing the results of six replicates prepared the same day at three different concentrations (200, 1000, and 2000 µg/kg). The procedure was repeated to determine inter-day precision by comparing results from samples prepared and analyzed on three different days. Coefficients of variation (CV, %) and standard deviations (SD) were calculated for each level. Percent recoveries were calculated as the measured content divided by the fortification level multiplied by 100. Matrix effects were calculated by comparing the slopes of calibration curves prepared by spiking blank feed samples and calibration curves in solvent. The uncertainty (U) was calculated as the ratio of the coverage factor (k = 2) and standard deviation (SD) of within-laboratory reproducibility and is expressed in percent.

U = k × SD within-laboratory reproducibility(1)

## 4. Conclusions

A simple qualitative and quantitative method for the simultaneous determination of five SAs from animal feed using HPLC-FLD was successfully developed and validated according to the European Commission Decision 2002/657/EC. The proposed method provided appropriate accuracy and precision and successfully analyzed different animal feeds. The good performance of this method satisfies the requirements of the detection of sulfonamides. This method can be used in multi-residue confirmation and quantification of sulfonamides in feeds.

## Figures and Tables

**Table 1:** Validation parameters of the high-performance liquid chromatography-fluorescence detection(HPLC-FLD) method.

Analyte	Recovery [%]			Repeatability [%]			Whitin-Laboratory Reproducibility [%]			CCα [µg/kg]	CCβ [µg/kg]	LOD [µg/kg]	LOQ [µg/kg]	U [%]
	Concentration Levels [µg/kg]			Concentration Levels [µg/kg]			Concentration Levels [µg/kg]							
	200	1000	2000	200	1000	2000	200	1000	2000					
Sulfaguanidine	79.3	102.1	89.9	7.8	8.7	3.0	11.8	11.5	11.1	265.2	315.2	34.5	41.3	24.4
Sulfadiazine	97.4	114.0	90.4	3.8	7.7	2.7	11.8	7.4	9.5	197.7	239.2	52.4	58.9	19.8
Sulfamerazine	103.4	103.0	93.5	5.7	8.0	5.9	6.1	10.0	9.7	224.5	263.2	63.5	68.4	21.2
Sulfametazine	103.9	103.5	91.7	4.6	6.5	6.1	5.9	14.9	10.9	266.7	326.9	79.5	89.9	20.3
sulfamethoxazole	94.8	102.2	100.9	5.9	7.9	9.1	8.3	14.2	10.1	274.6	337.9	39.7	43.1	23.3

**Table 2:** Gradient elution of sulfonamides with HPLC-FLD detection.

Time (min)	0.08% Acetic Acid in Milli-Q Water (A) (%)	Acetonitrile (B) (%)	Methanol (C) (%)
0–10	48	10	42
10–15	41	10	49
15–17	41	10	49
17–20	18	40	42
20–22	48	10	42
22–27	48	10	42
